# A dataset of rare copy number variants associated with neurodevelopmental and neuropsychiatric disorders

**DOI:** 10.1038/s41597-026-07345-6

**Published:** 2026-04-29

**Authors:** Alexandra Valeanu, Yuanyuan Duan, Javier Millán Acosta, Theo M. de Kok, Therese van Amelsvoort, Friederike Ehrhart

**Affiliations:** 1 Department of Translational Genomics, NUTRIM/MHeNs/GROW, Maastricht University, Maastricht, The Netherlands; 2 Department of Psychiatry and Neuropsychology, MHeNs, Maastricht University, Maastricht, The Netherlands

**Keywords:** Psychiatric disorders, Computer modelling

## Abstract

Copy number variations (CNVs) are large structural alterations of the genome that can contribute significantly to the genetic basis of neurodevelopmental and neuropsychiatric conditions, including schizophrenia, autism spectrum disorder, and intellectual disability. Although CNVs are genomically diverse, many result in overlapping clinical features and molecular changes. We present a curated machine readable dataset, *CNVPathwayAtlas*, that integrates 38 pathogenic CNVs with their genomic coordinates, affected genes, molecular pathways, associated syndromes, and phenotypes. Each CNV is linked to a curated molecular pathway providing mechanistic insight into affected biological functions. This dataset is integrated with external resources including WikiPathways, Orphanet, HGNC, and the Human Phenotype Ontology, and designed for compatibility with bioinformatics workflows. This dataset provides a structured foundation for analyzing the molecular effects of CNVs, and facilitates exploration of shared disorder mechanisms, diagnosis, identification of therapeutic targets, and drug discovery in neurodevelopmental and neuropsychiatric disorders.

## Background & Summary

Copy number variants (CNVs) are types of genetic variation involving DNA segments that are typically larger than 1 kilobase (kb). These structural alterations contribute both to human genetic diversity and to disease by affecting gene dosage, disrupting gene functions, or altering regulatory elements^1,2^. Among these variants, large and rare deletions/duplications contribute to the development of neurodevelopmental and neuropsychiatric disorders such as intellectual disability (ID), autism spectrum disorder (ASD), bipolar disorder (BD), and schizophrenia (SCZ)^3^.

The pathogenicity of CNVs depends not only on their size and genes but also on their inheritance and genomic context. Some CNVs arise *de novo*, spontaneously in the germline, while others are inherited, often showing variable expressivity and incomplete penetrance^4^. Often, certain recurrent CNVs, such as deletions at 22q11.2 or duplications at 16p13.11, are well-characterized risk loci for neuropsychiatric disorders^5^. However, many rare CNVs, especially those linked to very rare or syndromic conditions, remain poorly understood. Moreover, rare CNVs can affect early brain development and result in long term impairments in cognition, behavior, and social functioning^4,6^. As a result, CNVs contribute not only to individual morbidity but also to substantial economic and caregiving burdens on families and healthcare systems^7–9^.

Among neuropsychiatric conditions, schizophrenia has been one of the most intensively studied in terms of CNV burden. Although common variants identified through genome-wide association studies (GWAS) explain part of schizophrenia heritability^10,11^, a large proportion remains unexplained, referred to as the “missing heritability”^12^. Rare CNVs, each present in fewer than 0.5% of schizophrenia cases, are thought to account for part of this gap (^13^ and references therein). In population-based and clinical studies, CNVs are consistently enriched in individuals with schizophrenia, particularly in early-onset or treatment resistant cases. For example, in a study of Childhood Onset Schizophrenia (COS), defined as onset before the age of 13 years, approximatively 12% of individuals were found to carry at least one of 46 rare CNVs previously associated with adult-onset schizophrenia, autism spectrum disorder, intellectual disability, or epilepsy^14^.

Despite extensive research, bioinformatics resources lack detailed, clinically relevant CNV knowledge and are not readily accessible for data analysis. Existing initiatives such as Orphanet^15^, which provides structured data on rare diseases, and the Treatabolome project^16^, which links genetic diagnoses to therapeutic interventions, represent major advances in rare disease informatics. However, these resources have a broader scope and are not specifically focused on copy number variations.

To address this gap, we created a curated dataset focused on pathogenic copy number variations and their associated molecular pathways. The dataset integrates CNV summaries, gene-specific annotations, and linked syndromes in a structured and standardized format suitable for downstream bioinformatics analyses. It enables efficient retrieval of clinically and functionally relevant CNV information across genomic locations. Clinicians can use the dataset to review syndrome characteristics and gene content, while researchers and bioinformaticians can incorporate it into computational workflows. Ultimately, this dataset supports a systematic understanding, investigation, and interpretation of CNV syndromes.

## Methods

**Fig. 1:**
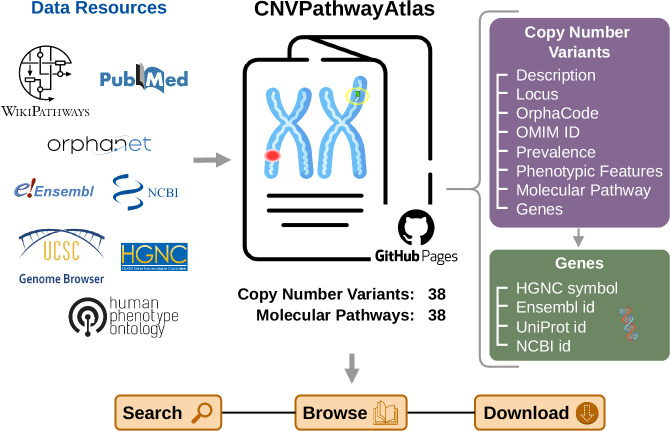
Overview of the *CNVPathwayAtlas* built with GitHub Pages, showing integrated data sources, number of curated CNVs and pathways, available user actions (search, browse, download), and the types of clinical and genomic information included for each CNV.

This section outlines the workflow we employed to generate the copy number variation dataset. The overview of the workflow is illustrated in Fig. 1.

### CNV selection

We selected rare pathogenic CNVs based on the reports by Marshall *et al*. (2017)^5^, and on the availability of curated molecular pathways in the WikiPathways Rare Diseases Community^17^. The CNVs included in this dataset are associated with neurodevelopmental and neuropsychiatric syndromes, and many are linked to schizophrenia. However, the dataset is not limited to schizophrenia-associated CNVs and can be easily expanded as new CNV pathways are made available.

Genomic coordinates follow the GRCh37.p13 reference genome, as reported by Marshall *et al*. (2017)^5^. To ensure compatibility with current resources, gene symbols were updated to the latest HGNC annotations, consistent with GRCh38. Corresponding GRCh38 coordinates for each CNV region were obtained using the UCSC LiftOver tool via the UCSC Genome Browser^18,19^. Gene annotations were retrieved using BioMart^20^, the UCSC Genome Browser^18^, and the HGNC database^21^. Additional gene metadata (e.g., Ensembl, NCBI Gene, and UniProt identifiers) were obtained from HGNC via programmatic queries.

### Molecular Pathway Construction

Molecular pathways were constructed using PathVisio^22^, a biological pathway editing and annotation tool. Integration of external identifiers was performed using BridgeDb^23^, enabling consistent mapping of gene products and metabolites. For each CNV, protein-coding genes within the deletion or duplication region were first retrieved using BioMart and PyEnsembl (github.com/openvax/pyensembl). Genomic coordinates were initially mapped to GRCh37.p13, then converted to GRCh38 for consistent annotation. Gene identifiers were cross-referenced and harmonized using MyGene.info^24^. Interaction data were gathered from several databases, including STRING^25^, BioGRID^26^, SIGNOR^27^, GeneCards^28^, UniProt^29^, and IntAct^30^. Protein-complex information was integrated from ComplexPortal^31^. Each pathway includes different interaction types, e.g. stimulation, inhibition, binding, and conversion, enabling representation of functional relationships.

### Data Collection and Processing

**Fig. 2:**
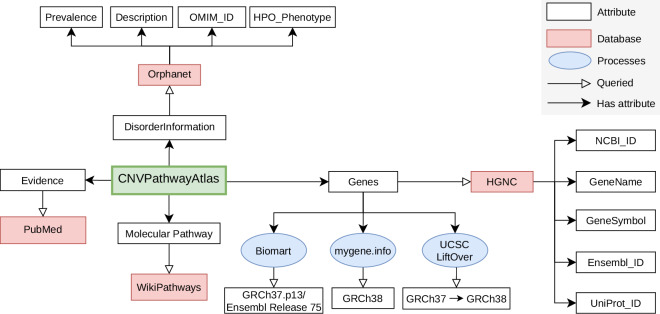
Data flow diagram showing integration of genomic, phenotypic, and pathway data into the CNVPathwayAtlas dataset. Databases queried include Orphanet, HGNC, PubMed, and WikiPathways. CNV genomic coordinates retrieve genes via BioMart and MyGene.info. USCS LiftOver tool is used to map genomic coordinates between different assemblies. Gene metadata and stable IDs come from HGNC, cross-referenced with Ensembl, NCBI, and UniProt. Disorder and OMIM associations are sourced from Orphadata, with OMIM IDs mapped through its cross-references. Phenotypic annotations derive from Orphadata and HPO. CNV evidence is collected from PubMed, with PMIDs linked to each CNV entry.

We developed a Python-based pipeline, hosted on GitHub (github.com/CNVPathwayAtlas), to process the CNV dataset. The pipeline takes as input a curated Excel file listing pathogenic CNVs along with relevant annotations including genomic loci, definition, evidence, associated genes, OrphaCodes, syndrome descriptions, and WikiPathways IDs. Gene-related data is retrieved from the HGNC database. This consists of approved gene symbol, HGNC ID, Ensembl ID, Entrez Gene ID, and UniProt ID. Syndrome-related metadata, i.e. deletion or duplication, for each CNV were sourced from Orphadata files^32^. The information includes definition, prevalence estimates and phenotypic features. The phenotypic features are classified according to Orphanet frequency categories: obligate (100%), very frequent (99–80%), frequent (79–30%), occasional (29–5%), very rare (<4–1%), and excluded (0%)^32^. Each syndrome was linked to its corresponding Orphacode^33^. Human Phenotype Ontology (HPO) terms and OMIM IDs were added based on curated mappings from Orphadata. Gene-pathway associations were retrieved via the WikiPathways API. A detailed overview of the data collection can be seen in Fig. 2.

## Data Records

The dataset has been deposited on Zenodo with the CC BY 4.0 license in both XLSX and JSON formats under the names CNVPathwayAtlas-data.xlsx and CNVPathwayAtlas-data.json^34^. The data are organized in a tabular structure with the following fields: CNV name, locus, chromosome, genomic start and end coordinates, region coordinates for both GRCh37 and GRCh38 builds, description, PubMed identifiers, gene annotations (HGNC symbol, HGNC name, HGNC ID, Entrez ID, Ensembl ID, UniProt ID), molecular pathway identifiers (WikiPathways), and Orphanet-linked attributes (ORPHAcode, cause, definition, prevalence, phenotypic features stratified by frequency, HPO ID, and OMIM ID). For programmatic access, the dataset is additionally provided in JSON format as a CNV-centric hierarchical structure, where each object represents a copy number variation and its associated annotations.

## Technical Validation

Data quality was assessed through manual curation to ensure accuracy, consistency, and biological validity of the dataset.

- Genomic mapping validation: Gene content within each CNV region was validated against both Ensembl and the UCSC Genome Browser to confirm correct mapping to genomic coordinates and consistency with the selected genome build.
- Cross-source verification: Gene content, syndrome associations, and phenotypic annotations were cross-checked against their original sources, including Orphanet, OMIM, and relevant literature. CNV entries were retrieved and compared across multiple iterations.
- Identifier consistency checks: Gene, pathway, and disease identifiers were validated for consistency across sources, ensuring correct cross-referencing and removal of ambiguous or outdated identifiers.
- Pathway mapping validation: Gene-pathway associations were validated by confirming that all mapped genes are present within the referenced pathways, ensuring that CNV-pathway links are biologically coherent.
- Phenotype annotation quality control: Phenotypic features and their frequency categories were checked for consistency and correct classification according to Orphanet definitions and to the HPO identifiers.
- Reproducibility: All data processing and integration steps were performed using scripted workflows, enabling reproducibility of the dataset from the original sources.

## Usage Notes

This dataset is intended for researchers studying neurodevelopmental and neuropsychiatric disorders. Upon downloading the complete dataset, users can construct knowledge graphs and perform network analyses. Example scripts for network construction and analysis are available on GitHub (github.com/CNVPathwayAtlas/cnv-usecase). The dataset can be used to identify phenotypes recurring across multiple CNV syndromes, investigate shared molecular mechanisms, and explore genes common to multiple pathways associated with distinct CNVs. Users can also interact with an updated version of the dataset through the *CNVPathwayAtlas* website (cnvpathwayatlas.github.io/cnv-website/), which provides an interactive interface for browsing and searching CNVs, pathways, and associated metadata. Each CNV has an individual page with detailed information, and comprehensive documentation is available to support data interpretation and exploration of potential applications.

## Data Availability

The dataset has been deposited on Zenodo under a CC BY 4.0 license^34^. It is available in both XLSX and JSON formats as CNVPathwayAtlas-data.xlsx and CNVPathwayAtlas-data.json. The resource integrates CNV-level genomic annotations, gene-level information, molecular pathway links from WikiPathways, and disease and phenotype annotations curated from Orphanet and mapped to Human Phenotype Ontology identifiers.

## Code availability

The source code for data processing is available at github.com/CNVPathwayAtlas.
